# Comparison of Two Canal Preparation Techniques Using Mtwo Rotary Instruments

**Published:** 2011-11-15

**Authors:** Faeze Hamze, Kiamars Honardar, Kiumars Nazarimoghadam

**Affiliations:** 1. Department of Operative Dentistry, Dental School, Kerman University of Medical Sciences, Kerman, Iran.; 2. Department of Endodontics, Shahed University of Medical Sciences, Tehran, Iran.

**Keywords:** Curvatures, Instrumentation, Nickel-Titanium, Root Canal Preparation

## Abstract

**INTRODUCTION:**

Root canal preparation is an important process in endodontic therapy. Nickel-titanium (NiTi) rotary file system can be used in single length technique (simultaneous technique) without early coronal enlargement, as well as in crown-down method. The purpose of this in vitro study was to compare single length with crown-down methods’ shaping ability using Mtwo NiTi files.

**MATERIALS AND METHODS:**

Fifteen acrylic-resin blocks containing simulated canals were divided into two experimental groups. In group A, single length technique was used and in group B root canals were prepared by crown-down technique. Pre- and post-preparation canals were photographed in a standardized manner and were superimposed. The inner and outer walls of canal curvature were evaluated at three points (apical, middle and coronal) to determine the greatest change. The data was statistically analyzed using the Student t-test by Statistical Analysis System (SAS) software.

**RESULTS:**

Statistical analysis revealed that in group B, dentine was equally removed within the canal coronal to the curvature, whereas in group A, the inner wall was predominantly removed (P<0.01). The two groups had no significant difference at the apical and middle points of the canal curvature.

**CONCLUSION:**

Our in vitro study revealed no significant difference between the single length method and crown-down technique using Mtwo for preparation of apical and middle portion of canal curvature.

## INTRODUCTION

Root canal preparation using nickel-titanium (NiTi) rotary system is a great advance in endodontics [1]. It had been proven that the root canal preparation using rotary (NiTi) files can significantly minimize the time required for canal instrumentation with minimal deviation from the original canal path compared with hand instrumentation. The design features of endodontic instruments are important and may have a significant effect on the cleansing efficiency of the instrument [2].

One of the most successful NiTi rotary systems is Mtwo. The cross-section shape of Mtwo is an "italic S" with two cutting blades. The rake angle of Mtwo is one of the most effective measures in NiTi rotary instruments, enhancing the cutting efficiency of this instrument. The tip is non-cutting, and the variable helical angle reduces the tendency of the instrument to be sucked down into the canal [3].

Mtwo instruments are used in a single length technique without early coronal enlargement. Each instrument is used up to the working length without apical pressure. Just when a tight contact is sensed by a clinician, the instrument is withdrawn 1-2mm so that it can be worked with a brushing action. In this way, the file will selectively remove the interferences and advance towards the apex. The instruments are used with a lateral pressing movement in order to obtain a circumferential cut [4]. Plotino et al. showed that the fatigue life of Mtwo instrument was reduced with a lateral brushing or pressing movement [5].

On the other hand, most rotary techniques require a crown-down approach to minimize torsional loads and to reduce the risk of instrument fracture [6]. Buchanan argued that applying conventional step back technique for greater taper files, in certain root forms, resulted in broken file in the apical regions of the root canal [7]. He introduced the crown-down method to virtually eliminate the risk of canal ledging and file breaking within the apical third of the canal [7]. Accordingly, all NiTi rotary systems on the market today have been developed based on the crown-down technique except the Mtwo system [1]. Moreover, comparing to the step back technique, crown-down preparation technique has some benefits such as less canal transportation [8][9], less post treatment pain [10], and less crack propagation in instruments [11].

The aim of the present in vitro study was to compare the ability of crown-down method to single length technique in maintaining the original shape of curved canals during preparation.

## MATERIALS AND METHODS

Fifty acrylic-resins simulated root canal blocks (VDW Co., Munich Germany) were used in this experimental study. The degree of curvature was 60º; the diameter and the taper of all simulated canals was equivalent to an ISO standard size #8 (0.02 taper) root canal instrument.

Simulated canals were divided into two groups, each group consist of 25 blocks. The blocks were fixed on a holder during preparation. At first stainless steel file #10 (Mani Co., Tokyo, Japan) was introduced to canals. Prior to file usage, files were coated with chelating agent File Care (VDW Co., Munich, Germany) and copious irrigation with tap water was performed repeatedly after using each file. The following preparation sequence was used for experimental groups:

### Group A:

For coronal enlargement, IntroFile orifice shaper (VDW, Munich, Germany) enlarged the orifice for 5 seconds. Then Mtwo (VDW Co., Munich, Germany) #10 (0.04 taper), #15 (0.05 taper), #20 (0.06 taper) and #25 (0.06 taper) were used respectively to the full length of canal, each file was rotated for 3 seconds in the canal until it reached the apical point.

### Group B:

Same as group A, IntroFile orifice shaper was implemented for coronal enlargement for 5 seconds. Mtwo #25 (0.06 taper) was worked passively through the coronal third, then #20 (0.06 taper) passively enlarged the middle part and #15 (0.05 taper) in the apical third of the canal. For final shaping of canals, Mtwo #25 (0.06 taper) were used to full working length; each file was rotated for 3 second.

All files were used by the torque controlled motor and rotational speed programmed in the file library of the Endo IT motor (VDW, Munich, Germany). NiTi instruments were only used to prepare four canals; the preparation was carried out by a single operator.

**Figure 1:**
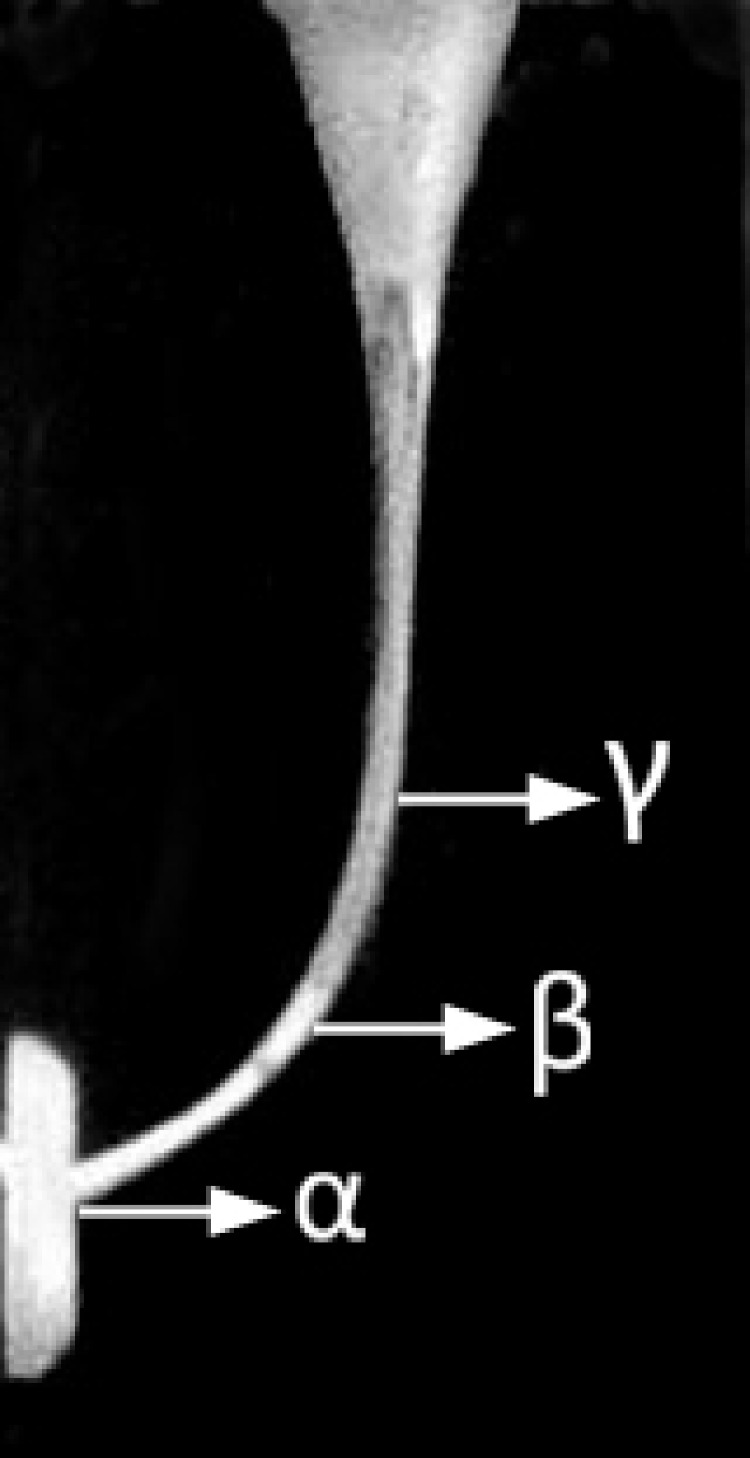
Both sides of the artificial canals were evaluated at three points

Pre- and post-preparation canals were photographed in a standardized manner (Mavo Zoom ×50) and stored in a computer. By means of adobe Photoshop element 8 software, pre and post images were superimposed to determine the changes in canal configuration resulting from preparation process. This was performed by evaluating the canals’ walls on three points, the first 1mm from the apex (named as α), the second at the middle (named as β) and the last the most coronal part of the curvature (named as γ) (Figure 1). At each point, with ±0.1mm precision, the amount of resin removed at the inner or outer sides was recorded.

The data was statistically analyzed using the Student’s t-test by Statistical Analysis System (SAS) software (version 9.1).

## RESULTS

According to this study, groups A and B have no statistically significant difference at α and β points (Table 1). P-values at α and β points are 0.81 and 0.37, respectively.

**Table 1:** Mean values and Standard Errors of resin removal in 25 different groups at three points of canal wall

**Evaluated Point of canal**	**α point**		**β point**		**γ point**	
**Groups**	**A**	**B**	**A**	**B**	**A**	**B**
**Mean±Std Err**	0.32±0.095	0.4±0.1	0.36±0.098	0.36±0.081	0.04±0.04	0.16±0.074

Nevertheless, Student’s t-test revealed that these two groups had statistically significant difference at γ point; more resin was removed from the inner wall in group A. In group B both sides of the canal were equally removed.

This means that in group B the canals’ internal shape remained more unchanged at the most coronal part of the curvature, even though these two groups had no difference at the other sections of the canal curvature (P<0.01).

## DISCUSSION

This study was carried out with the aim of assessing the preparation of rotary Mtwo NiTi instrument using crown-down technique with single length technique [4]. Nonetheless, our null hypothesis is rejected because no significant difference was obtained between two shaping methods.

The present study compared the preparation abilities of the instruments under strictly controlled laboratory conditions, using clear resin blocks with the same curvature and angle. Use of simulated canals in resin blocks does not reflect the action of the instruments in root canals of human teeth because of differences in the surface texture, hardness and cross section. However, resin blocks allow a direct comparison of the preparation ability of different instruments [12].

In the current study, Mtwo rotary file with single length technique was used; the advantages included: respect the canal anatomy [13] without substantial change in working length [14], significantly better debris removal comparing to K3 and Race systems [15], and also less canal zip than K3 or ProTaper instruments [16]. On the contrary, too little information exists about the performance of Mtwo files as a crown-down system.

Canal preparation is a critical aspect of endodontic treatment because it influences the outcome of the subsequent treatment phases [17]. One of the most important goals of endodontic preparation is to shape the root canal without deviating from the original canal position [18]. Consequently, if the inner and the outer walls of the canals are equally removed it creates more anatomic form; this is what we observed in group B. In this group, crown-down technique mostly followed the internal anatomy at the most coronal part of the canal curvature. Conversely, at this part of the canal, the curvature’s inner wall was predominantly removed using single length technique; this concurred with Schafer et al.’s study [14]. The γ point in our study is equivalent with the point that is 8mm distant from the apex in Schafer et al.’s investigation. It would be due to the brushing movement of Mtwo files in single length technique which is applied as soon as a tight contact is being sensed [4].

According to the shape of the simulated canal, the beginning point of the curve, which is described as the most coronal part of the canal curvature, is of the most important sites that would produce a binding sensation with high cyclic fatigue. Therefore, based on the cross-section of file with positive rake angle, greater removal would occur on the inner wall of the root canal.

In our study there was no significant difference between crown-down and single length technique at the three different points of acrylicresin blocks with simulated canals. At α point, the apex anatomy was respected; this is consistent with Veltri et al. who reported keeping-centered preparations in the apical region using Mtwo instrument [19].

No fractured with Mtwo instruments occurred. This finding is in accordance with previous researchers such as Schafer et al and Veltri et al. [14][19]. But Bürklein et al. reported one Mtwo instrument separation during the enlargement of 28º curved simulated canals [20]. The main cause of no Mtwo file fracture even after enlargement of four canals may be due to its increasing pitch length from the tip to the shaft of these instruments or less usage. As already reported, a varying pitch length along the working part of the instrument reduces the tendency of the file to screw-in [21] mini-mizing the risk of instrument fracture [14].

Goerig et al. described a step-down technique of radicular access [22]. He concluded that there were various advantages to this method. First, the vast majority of microorganisms and pulpal tissues were removed early during cleaning and shaping, thereby reducing the potential for extruding material into the periapical area. Second, instruments pass unhindered into the apical area once the coronal two-thirds are enlarged. Last, the methodology allows for better penetration of the irrigant [22]. The crown-down technique uses step-down concepts for shaping the entire canal length. It is a biologically ideal shaping method because of its control over the movement of contents [18].

Since numerous advantages of crown-down technique has lead to it’s popularity in rotary systems [6][7] and because dentists are more familiar with rotary crown-down operation, if we could use Mtwo system by crown-down manner it would be more user friendly and biologically accepted.

## CONCLUSION

Although Mtwo could be used in single length technique, our study revealed no significant difference between this method and crown-down technique using Mtwo for preparation of apical and mid portion of canal curvature. However, at the most coronal part of canal curvature, the crown-down technique better respected the canal anatomy.

Perhaps after additional studies, maybe Mtwo system could be used by crown-down technique which is not only more beneficial but also more users friendly.
